# Supplementary material for: Organic flash memory on various flexible substrates for foldable and disposable electronics
Source: Nat Commun. 2017 Sep 28;8:725. doi: 10.1038/s41467-017-00805-z (PMC5620045; doi:10.1038/s41467-017-00805-z)
Supplement: Supplementary file 1 — Supplementary Information [file 41467_2017_805_MOESM1_ESM.pdf]

Supplementary Figure 1.

Effect of dielectric constant (*k*) on the current flow across the blocking or tunneling dielectric layer (BDL or TDL). Current density (J_i) vs. applied voltage (V_i) of metal/insulator/metal (MIM) devices with 'I' being **a)** 48 nm-thick pEGDMA, and **b)** 8.5 nm-thick pV3D3. **c)** The expected values of J_i for BDL and TDL vs. V_{CG} of a M/BDL/M/TDL/M device wherein M is Al, BDL is the 48 nm-thick pEGDMA, and TDL is the 8.5 nm-thick pV3D3. V_{CG} was converted from the coupling ratio (α_{CR}) for each case of BDL and TDL. For more detailed description on coupling ratio, refer to Supplemtary Note 1.

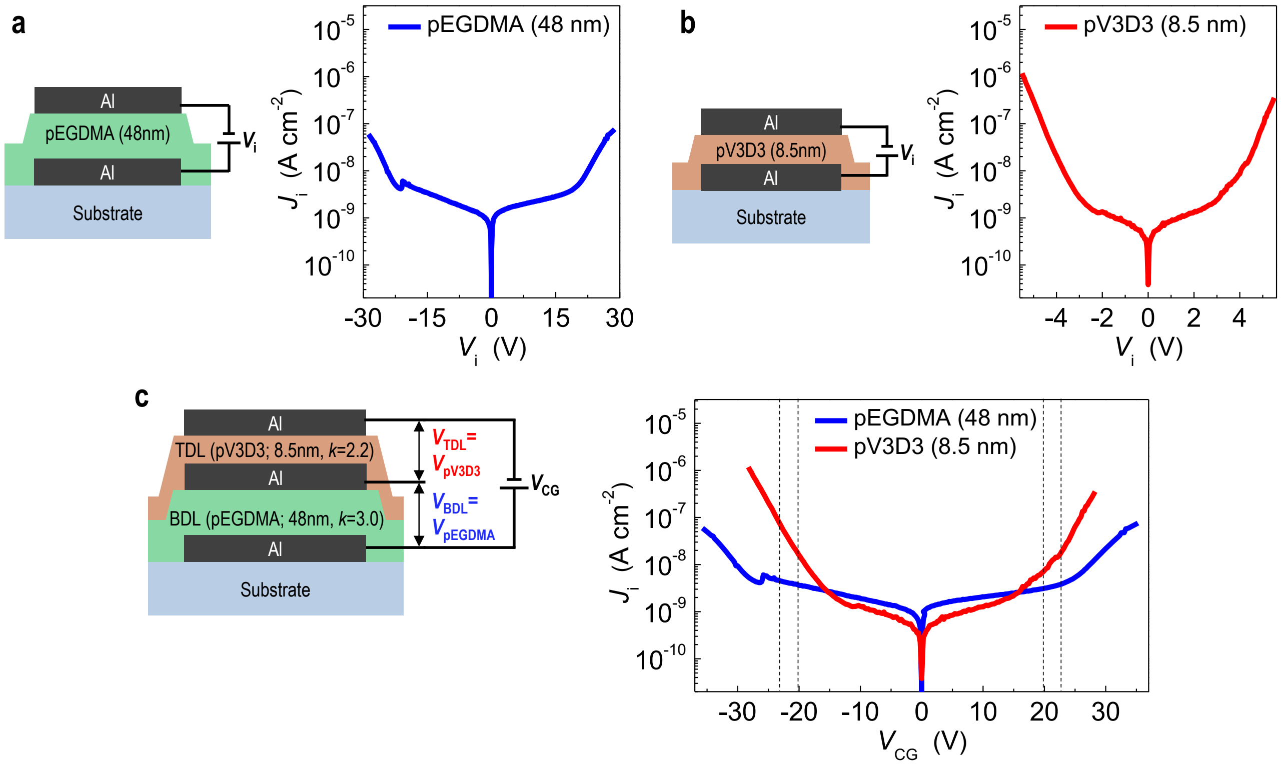

Supplementary Figure 2.

Cycling endurance characteristics of flexible organic flash memory fabricated on a PET substrate. Memory characteristics vs. the number of repetitive Programming/ Erasing operations up to 1,000 times: **a)** transfer memory curves and **b)** threshold voltage (V_{th}) distribution. **c)** Measurement procedure for cycling endurance test by repetitive electrical stress ($\tau_{P/E}$= 1 s). Data are color-coded; 'red' indicates data or operations related to erasing and 'blue' indicates those related to programming. Gate leakage current after the 1,000 cycles is also shown in **a**) (dotted line)

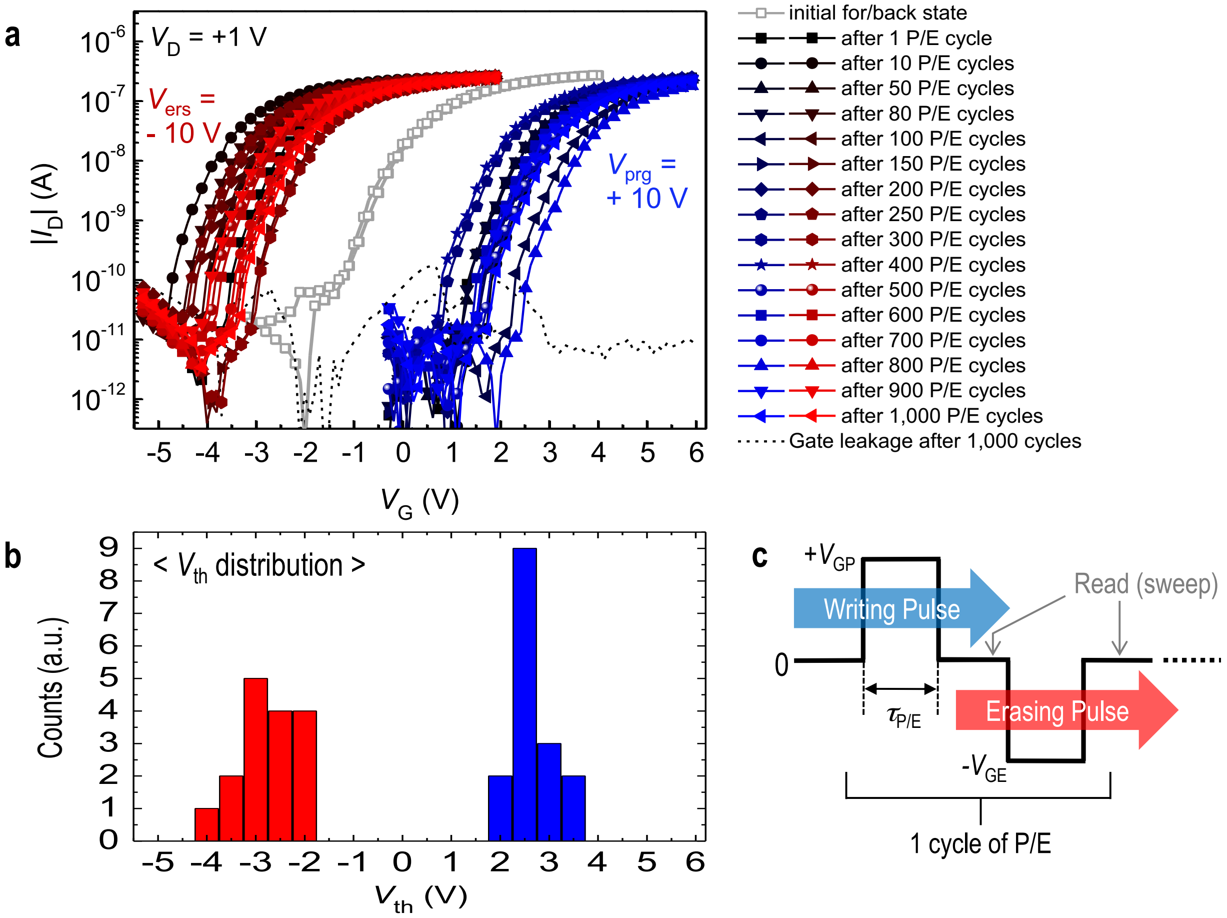

Supplementary Figure 3.

Memory characteristics vs. flexural tensile strain. **a)** Transfer memory curves of a flexible organic flash memory on a 250 μm-thick PET film. Gate leakage current (I_G) after 2.8 % strain is also shown (dotted line). **b)** J_i-E_i characteristics of of a pEGDMA-based MIM device obtained under tensile strain of 0.6 % to 4.0 %. **c)-j)** Photographs of the flexible organic device on a 250 μm-thick PET film under various levels of strain.

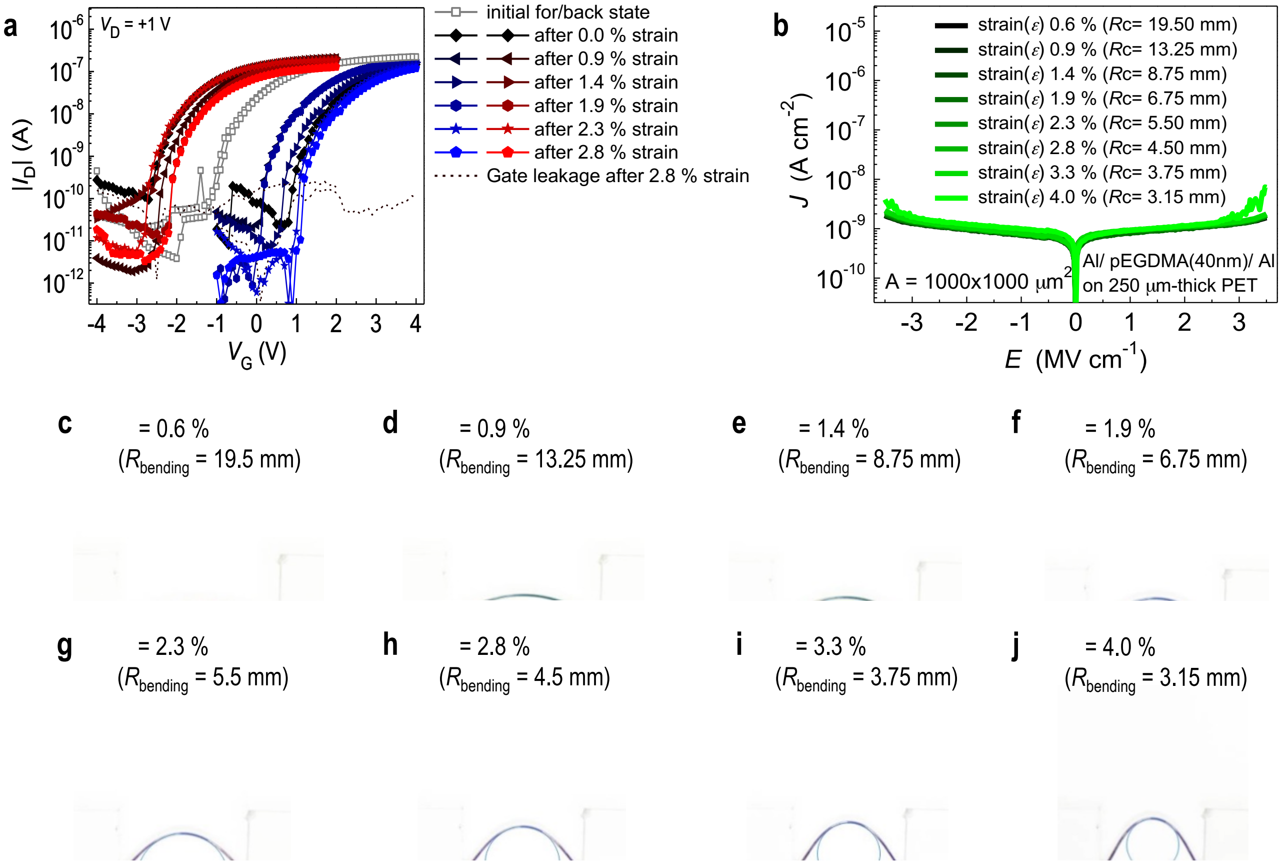

Supplementary Figure 4.

Characteristics of the organic flash memory fabricated on a 100 µm-thick PET substrate after repeated bending cyles.: **a)** transfer curves showing consistent operations over various bending cycles at flexural strain of 1.1%. Low gate leakage current (I_G) after 10,000-times bending is shown as a dotted line; **b)** threshold voltage (V_{th}) distribution. **c)** a photograph of the flexible organic flash memory fabricated on a 100 µm-thick PET film under bending test.

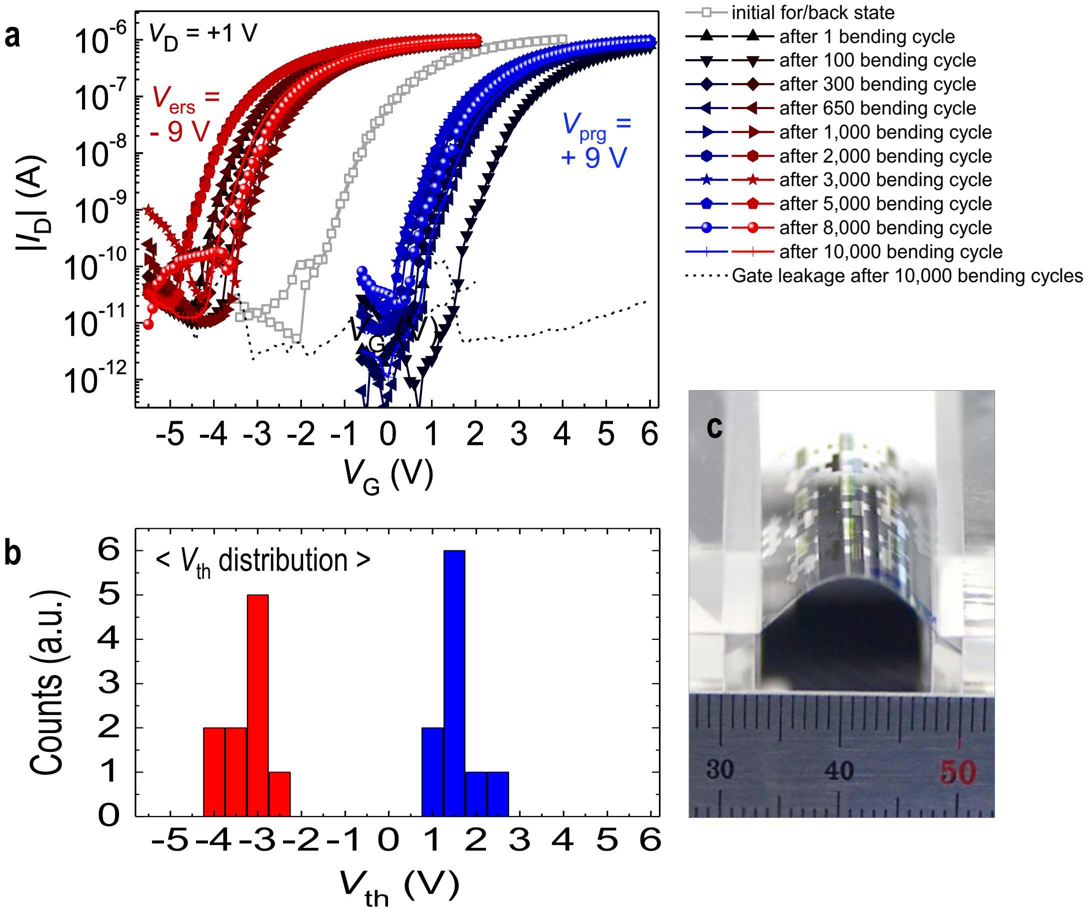

Supplementary Figure 5.

Flexible C_{60}-based thin-film transistor (TFT) characteristics: TFT performance vs. flexural strain. a) OTFT structure with pV3D3 and pEGDMA used to test the flexibility of TFT devices. **b)** Transfer curves of a flexible OTFT on a 250 μm-thick PET film measured for flexural strain of 0 % to 4.0 %. **c)-f)** Device characteristics of the C_{60}-based TFT obtained for various strain values up to 4% : **c)** gate leakage current (I_G) , **d)** hysteresis (=|$V_{G,forward}$ @ I_D= 100 nA - $V_{G,backward}$ @ I_D= 100 nA|), **e)** threshold voltage (V_{th}), and **f)** saturation mobility degradation ($\mu_{sat}/\mu_{sat,o}$). Degradation is shown to be not siginifcant until the strain of 3.3 % is applied.

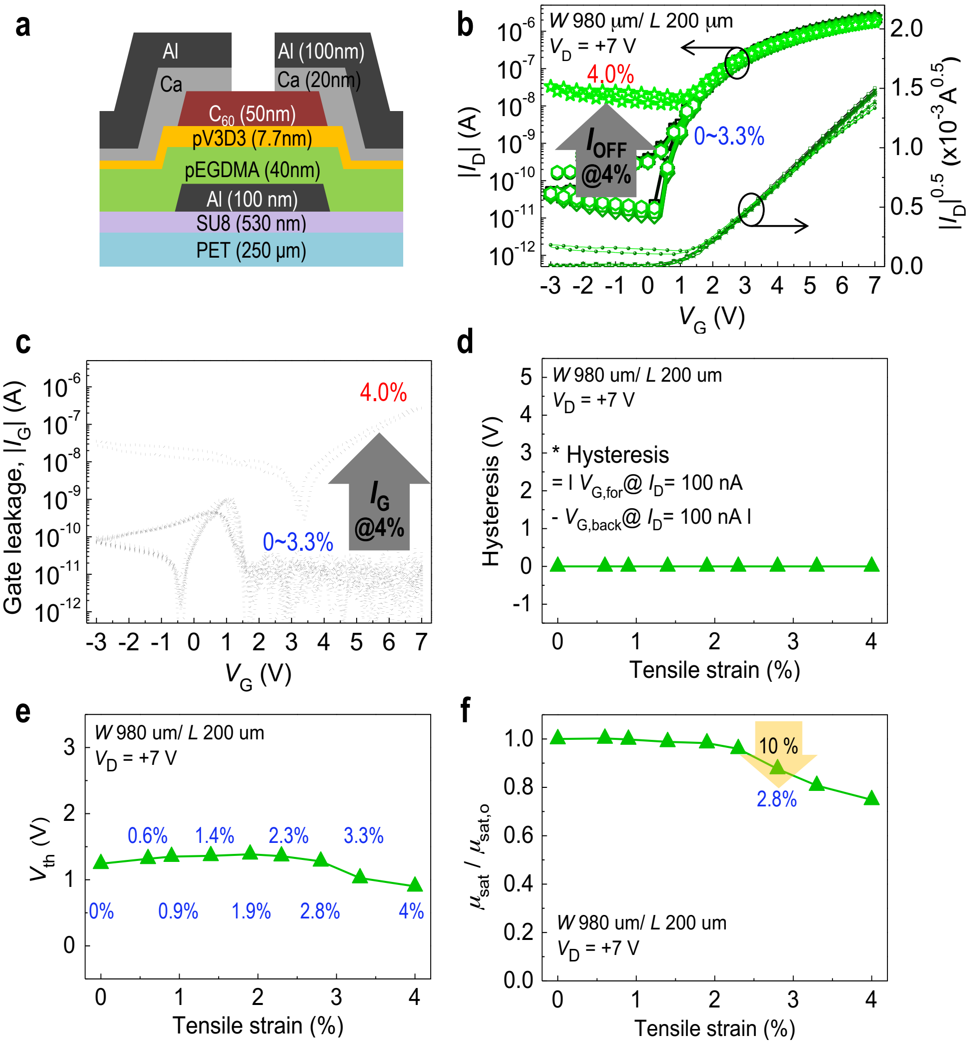

Supplementary Figure 6.

Custom-built test setup for characterization of foldable memory devices. The testing assembly is based on a test fixture and a slider equipped on a computer-controlled motorized translator, whose speed can be varied from a few μm s^{-1} to a few cm s^{-1}. The test fixture consists of a sample loader, a rigid piano wire, and two wire presses, one of which contains a tuner mechanism used in a guitar for proper tension management. To minimize the handling issue of ultrathin devices on Mylar™ substrates, foldability test process is carefully done as follow: **a)** setting up the components of the test fixture, **b)** sliding in one end of a sample underneath the wire and attaching that end on the loader using a PET-tape, **c)** folding the sample over the wire and attaching the opposite end of the sample on the slider via a long extended Kapton tape. The slider is then translated back and forth to fold or unfold a device against the piano wire. (See Supplementary Video 1 and 2 to see the folding test operation in action.) Example pictures of half-released and fully folded states are provided as insets. **d)** Photograph of the test assembly integrated with a manual probe station. e) The enlarged version of Fig. 3e in the main text.

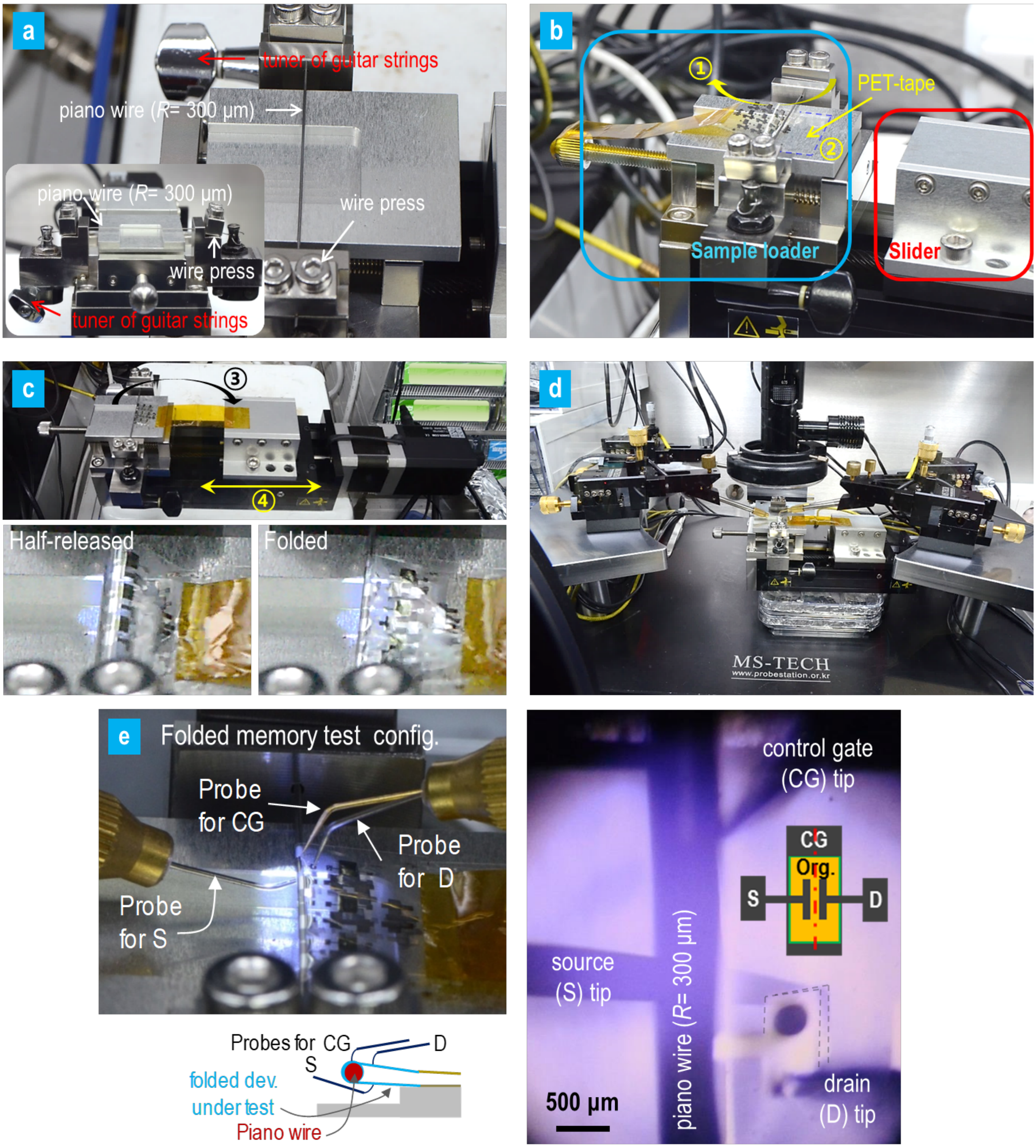

Supplementary Figure 7.

Comparison of the present work with the TFT-based non-volatile memory devices reported in the literature. The graph classifies the devices according to the kinds of BDL/TDL layers; inorganic or organic ones. Those with 'ε' values correspond to the performance data of flexible memory devices. In those cases, 'ε' refers to the maximum strain tested in the corresponding work. The points in the figure is generated using the date in Supplementary Table 2, and the numbers in square brackets refer to reference numbers in the main text.

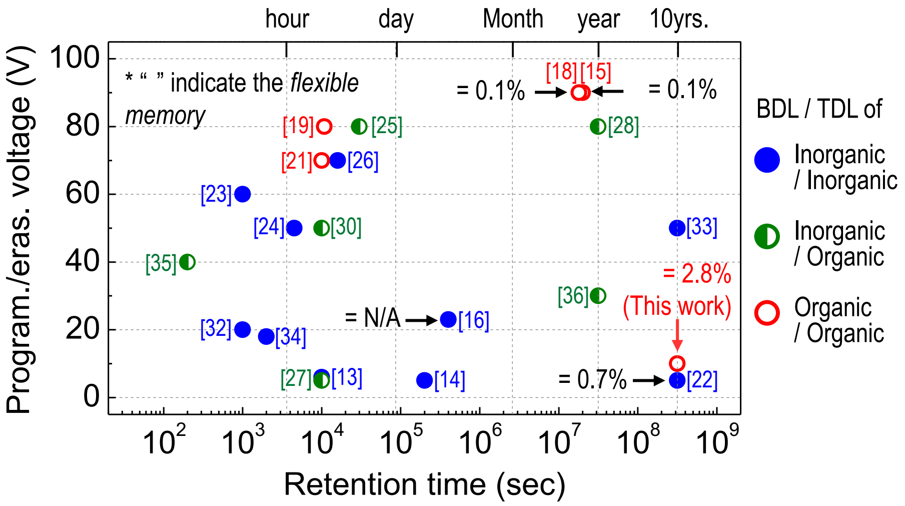

Supplementary Figure 8.

Planarization of paper with iCVD grown polymers effect on paper for disposable organic flash memories. Scanning electron microscope (SEM) images obtained for the top surfaces of the following: **a)** a pristine dye-sublimation paper (DP), **b)** 1.5 µm-thick poly(divinylbenzene) (pDVB) layer on the pristine DP, **c)** DP/ Al/ pEGDMA/ Al/ pV3D3/ C_{60}, and **d)** DP/ pDVB(1.5µm)/ Al/ pEGDMA/ Al/ pV3D3/ C_{60}.

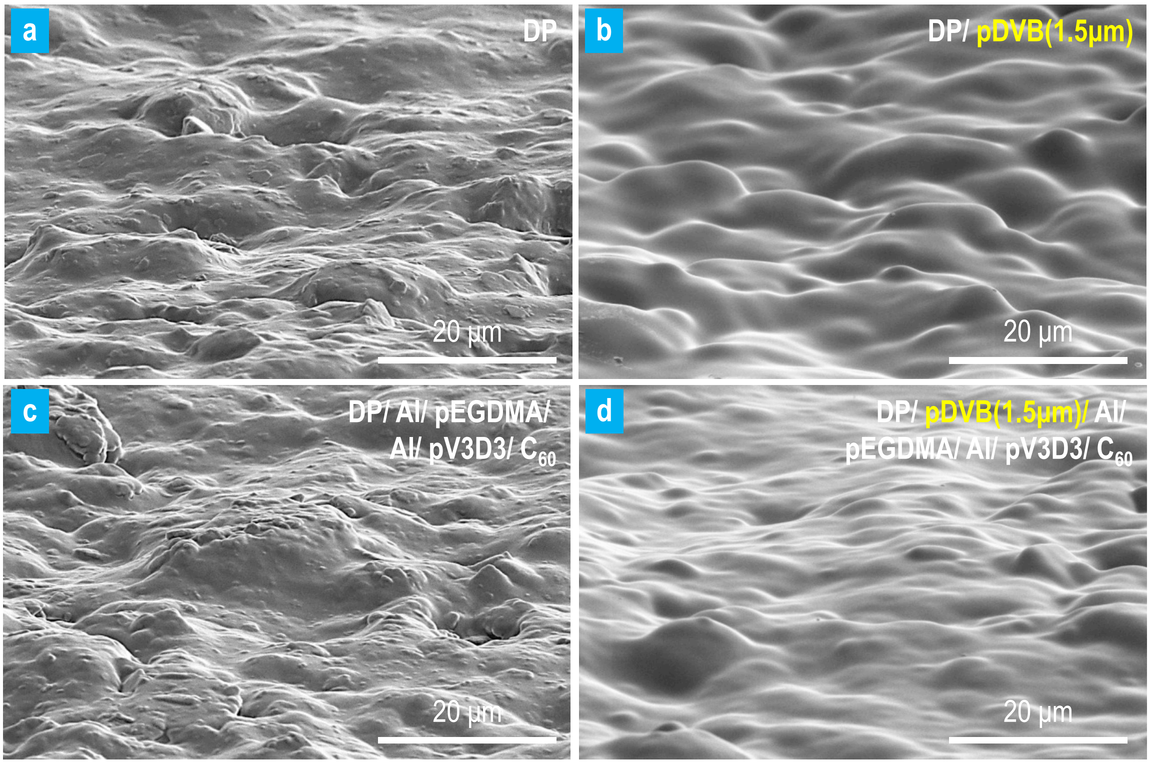

Supplementary Table 1.

The net voltage across a layer of interest for a given V_{CG} in the proposed memory vs. operation conditions.

State	Layer	$V_{\mathrm{layer}}^{\mathrm{(total)}}$ (=the total voltage across a given layer for V_{CG})
initial	BDL (pEGDMA)	$V_{\mathrm{bi,pEGDMA}}$
	TDL (pV3D3)	$V_{\mathrm{bi,pV3D3}}$
	Channel (C_{60})	~ 0
Programmed (**P$_0$**)	BDL (pEGDMA)	$V_{\mathrm{bi,pEGDMA}} + \Delta V_{\mathrm{th,pEGDMA}}^{\mathrm{P_0}}$
	TDL (pV3D3)	$V_{\mathrm{bi,pV3D3}} + \Delta V_{\mathrm{th,pV3D3}}^{\mathrm{P_0}}$
	Channel (C_{60})	$\Delta V_{\mathrm{th},C_{60}}^{\mathrm{P_0}}$
Erased (**E$_0$**)	BDL (pEGDMA)	$V_{\mathrm{bi,pEGDMA}} + \Delta V_{\mathrm{th,pEGDMA}}^{\mathrm{E_0}}$
	TDL (pV3D3)	$V_{\mathrm{bi,pV3D3}} + \Delta V_{\mathrm{th,pV3D3}}^{\mathrm{E_0}}$
	Channel (C_{60})	~ 0
Being programmed (**Prg**.)	BDL (pEGDMA)	$\left(V_{\mathrm{bi,pEGDMA}} + \Delta V_{\mathrm{th,pEGDMA}}^{\mathrm{E_0}}\right) + (1-\alpha_{\mathrm{CR}}^{\mathrm{prg}})V_{\mathrm{CG}}^{\mathrm{prg}}$
	TDL (pV3D3)	$\left(V_{\mathrm{bi,pV3D3}} + \Delta V_{\mathrm{th,pV3D3}}^{\mathrm{E_0}}\right) + \alpha_{\mathrm{CR}}^{\mathrm{prg}}V_{\mathrm{CG}}^{\mathrm{prg}}$
	Channel (C_{60})	~ 0
Being erased (**Ers**.)	BDL (pEGDMA)	$\left(V_{\mathrm{bi,pEGDMA}} + \Delta V_{\mathrm{th,pEGDMA}}^{\mathrm{P_0}}\right) + (1-\alpha_{\mathrm{CR}}^{\mathrm{ers}})V_{\mathrm{CG}}^{\mathrm{ers}}$
	TDL (pV3D3)	$\left(V_{\mathrm{bi,pV3D3}} + \Delta V_{\mathrm{th,pV3D3}}^{\mathrm{P_0}}\right) + \alpha_{\mathrm{CR}}^{\mathrm{ers}}V_{\mathrm{CG}}^{\mathrm{ers}}\beta$
	Channel (C_{60})	$\Delta V_{\mathrm{th},C_{60}}^{\mathrm{P_0}} + \alpha_{\mathrm{CR}}^{\mathrm{ers}}V_{\mathrm{CG}}^{\mathrm{ers}}(1-\beta)$

The table shows the total voltage across a given layer of interest ($V_{\mathrm{layer}}^{\mathrm{(total)}}$) expected in the proposed memory biased at V_{CG} for each of the device states: initial, programmed (**P$_0$**; $V_{CG} = 0$), erased (**E$_0$**; $V_{CG} = 0$), being programmed (**Prg.**; $V_{CG} = V_{\mathrm{CG}}^{\mathrm{prg}}$), or being erased (**Ers.**; $V_{CG} = V_{\mathrm{CG}}^{\mathrm{ers}}$). $V_{\mathrm{layer}}^{\mathrm{(total)}}$ reflects (i) the effect of built-in voltages ($V_{\mathrm{bi,\ layer}}$) owing to the initial Fermi level alignment in thermal equilibrium; (ii) the effect of the stored or erased charges that appear as threshold voltage shift ($\Delta V_{\mathrm{th,layer}}^{\mathrm{P_0}}$ or $\Delta V_{\mathrm{th,layer}}^{\mathrm{E_0}}$); and (iii) that of the applied bias influenced by the coupling ratio, $\alpha_{\mathrm{CR}}^{\mathrm{prg}}$ or $\alpha_{\mathrm{CR}}^{\mathrm{ers}}$. Note that, during the erasing operation, C_{60} channel is set to be off (i.e. insulating), and thus $V_{\mathrm{a,FG}}$ is not solely applied to TDL (pV3D3) but divided into pV3D3 and C_{60} with the ratio of $V_{\mathrm{a,\ pV3D3}}$ to $V_{\mathrm{a,C60}}$ (=β) given by:

$$\beta = \frac{V_{\mathrm{a,pV3D3}}}{V_{\mathrm{a},C_{60}}} = \frac{d_{\mathrm{pV3D3}}k_{C_{60}}}{d_{C_{60}}k_{\mathrm{pV3D3}} + d_{\mathrm{pV3D3}}k_{C_{60}}} \quad \cdots \quad (1)$$

It should also be noted that both which $\alpha_{\mathrm{CR}}^{\mathrm{ers}}$ differs from $\alpha_{\mathrm{CR}}^{\mathrm{prg}}$ because the effective area and thickness used for definition of capacitance (C) across FG/TDL/C_{60}/(D or S) are different as shown in Fig. 2c and 2d in the main text. They are given by the following equations:

$$C = \varepsilon_0 \frac{k_{\mathrm{material}} \times A_{\mathrm{effective-area}}}{V_{\mathrm{a},C_{60}}} \quad \cdots \quad (2)$$

$$\alpha_{\mathrm{CR}}^{\mathrm{prg}} = \frac{C_{\mathrm{BDL}}^{\mathrm{prg}}}{C_{\mathrm{BDL}}^{\mathrm{prg}} + C_{\mathrm{TDL}}^{\mathrm{prg}}} = \frac{C_{\mathrm{pEGDMA}}^{\mathrm{prg}}}{C_{\mathrm{pEGDMA}}^{\mathrm{prg}} + C_{\mathrm{pV3D3}}^{\mathrm{prg}}} \quad \cdots \quad (3)$$

$$\alpha_{\mathrm{CR}}^{\mathrm{ers}} = \frac{C_{\mathrm{BDL}}^{\mathrm{ers}}}{C_{\mathrm{BDL}}^{\mathrm{ers}} + C_{\mathrm{TDL}}^{\mathrm{ers}}||C_{\mathrm{channel}}^{\mathrm{ers}}} = \frac{C_{\mathrm{pEGDMA}}^{\mathrm{ers}}}{C_{\mathrm{pEGDMA}}^{\mathrm{ers}} + C_{\mathrm{pV3D3}}^{\mathrm{ers}}||C_{C_{60}}^{\mathrm{ers}}} \quad \cdots \quad (4)$$

$$\text{when } C_1||C_2 = \frac{C_1C_2}{C_1 + C_2}$$

Supplementary Table 2

Summary of the structure and performance of flash-type memory devices based on organic or emerging materials

**Lead Author[ref]**	**Subst-rate**	**Cha-nnel***	**BDL** (d_{BDL}in nm)**/ CSL/ TDL** (d_{TDL}in nm)†	ΔV_{th}, V_{op}§ (V, V)	t_{ret} (sec)	**Stra-in** (%)	Δn_{fg} (10^{12} cm^{-2})
**Organic flash memory devices on rigid substrates**							
Novembre, C. [24]	Si	Pen	SiO_2 (200)/Au NPs/ -	22, 50	4.5×10^3	-	2.4
Kim, S-J. [25]	Si	Pen	SiO_2 (100)/Au-NPs/ PMMA (40)	34, 80	3×10^4	-	7.3
Kim, Y-M. [26]	Si	Pen	SiO_2 (100)/[PE/Au-NPs]/ [HfO_2/PVP] (15/54)	14.6, 70	1.6×10^4	-	3.1
Chang, H-C. [27]	Glass	Pen	HfO/[CuPC NPs/ N-C_{60}]/ cPVP	4.4, 5	1×10^4	-	1.8
Park, Y. [28]	Si	Pen	SiO_2 (200)/Graphene/PS (15)	23, 80	~3.2×10^7	-	2.3
Yi, M. [29]	Si	Pen	SiO_2 (300)/Au-NPs/PMMA (25)	43, 150	9.0×10^3	-	2.9
Shih, C-C. [21]	Glass	Pen	cPVP (400)/[ZnO NPs/ PVPK]/-	60,70	1.0×10^4	-	3.4
Shih, C-C. [30]	Si	Pen	SiO_2 (100)/[PF & PFBT NPs]/PMAA (30)	35,50	1.0×10^4		7.4
Han, S-T. [33]	Si	Pen	SiO_2 (100)/[Metal NPs/ MoS_2 nanosheets]/ Al_2O_3 (5)	27.5, 50	>3.2×10^8		5.9
Baeg, K-J. [19]	Glass	F8T2	PVP (340)/Au-NPs/ PS (22)	30, 80	1.1×10^4		3.0
Liu, Z. [35]	Si	P3HT	SiO_2 (100)/Au-NPs/ PVP (10)	27, 40	2.0×10^2		5.8
Lee. S. [36]	Si	C_{60}	SiO_2 (50)/[Ag-NPs/d-SAM]/BCB (12)	4.1, 30	~3.2×10^7		1.8
**Organic flash memory devices on flexible substrates**							
Sekitani , T.[13]	PEN	Pen	[AlO_X/SAM (4/6)]/ Al/ [AlO_x/SAM (4/6)]	2, 6	1.0×10^4	-	8.1
Kaltenbrunner[14]	PEN	Pen	AlO_X (8.5)/Al/ [AlO_x (8.5)/SAM]	2.5, 5	2.0×10^5	-	1.6
Kim, S.-J. [15]	PES	Pen	PVP (400)/Au-NPs/ PVP (20)	15, 90	2.0×10^7	0.1	0.86
Kim S. M. [16]	PEN	Grap.	Al_2O_3 (25) /HfO_x/ Al_2O_3 (8)	6, 23	4.0×10^5	-	13
Kim, S-J. [18]	PES	Pen	PVP (400)/Au-NPs/ PVP (10)	10, 90	1.8×10^7	0.1	0.57
Han, S-T. [22]	PET	Pen	Al_2O_3 (30)/[Au-NPs/ rGO]/ Al_2O_3 (10)	2, 5	>3.2×10^8	0.7	3.6
**Lee, S. [This work]**	**PET**	**C_{60}**	**pEDGMA (40)/Al/ pV3D3 (14)**	**5, 10**	**>3.2×10^8**	**2.8**	**2.1**
**Flash memory devices based on emerging channel materials other than organic materials**							
Gupta, D. [23]	Si	ZnO	SiO_2 (100)/Ag-NPs/ -	28.5, 60	1.0×10^3	-	6.1
Lee, S. Y. [31]	Si	Grap	SiO_2 (300)/Graphene/ -	120, 150	1.0×10^4		8.6
Li, D. [32]	Si	BP	SiO_2 (300)/MoS_2/HBN (25)	60, 20	1.0×10^3		4.3
Bertolazzi S. [34]	Si	MoS_2	[Al_2O_3/HfO_2 (1/30)]/Graphene/ [Al_2O_3/HfO_2 (1/6)]	8, 18	2.0×10^3		**28**

* "Pen" = pentacene; "Grap"= graphene; "BP" = black phosphorous; "F8T2" = Poly(9,9-dioctylfluorene-alt-bithiophene); "P3HT" = Poly(3-hexylthiophene)

† Square brackets were used to indicate the case of multi-component BDL, CSL, or TDL, where CSL refers to a charge storage layer, which could be FG, metal nano-particles (NPs), and so on. Those without values in parentheses indicate that thickness values were not reported in the corresponding papers. "HBN" refers to hexagonal boron nitride.

§ V_{op} = the larger of V_{prog} and V_{erase} .

Supplementary Note 1.

Further description on coupling ratio. Let us consider an S/TDL/FG/BDL/CG structure shown in Supplementary Fig. 1c, which is equivalent to the memory structure underneath S electrode except for the absence of a semiconductor layer. Let us assume S, FG, and CG are all based on the same kind of metal for simplicity's sake. Under capacitive approximation, 'coupling ratio' (α_{CR}) or the ratio of the voltage between S and the FG across TDL ($=V_{FG}$) to V_{CG} and the electric fields across TDL and BDL (E_{TDL}, E_{BDL}) are then given by:

$$\alpha_{\mathrm{CR}} = \frac{V_{\mathrm{FG}}}{V_{\mathrm{CG}}} = \frac{1}{C_{\mathrm{TDL}}}\left(\frac{1}{C_{\mathrm{TDL}}} + \frac{1}{C_{\mathrm{BDL}}}\right)^{-1} = \frac{d_{\mathrm{TDL}}k_{\mathrm{BDL}}A_{\mathrm{BDL}}}{d_{\mathrm{TDL}}k_{\mathrm{BDL}}A_{\mathrm{BDL}} + d_{\mathrm{BDL}}k_{\mathrm{TDL}}A_{\mathrm{TDL}}} \quad \cdots (5)$$

$$E_{\mathrm{TDL}} = \frac{V_{\mathrm{TDL}}}{d_{\mathrm{TDL}}} = \frac{V_{\mathrm{FG}}}{d_{\mathrm{TDL}}} = \frac{\alpha_{\mathrm{CR}}V_{\mathrm{CG}}}{d_{\mathrm{TDL}}} = \frac{k_{\mathrm{BDL}}A_{\mathrm{BDL}}}{d_{\mathrm{TDL}}k_{\mathrm{BDL}}A_{\mathrm{BDL}} + d_{\mathrm{BDL}}k_{\mathrm{TDL}}A_{\mathrm{TDL}}}V_{\mathrm{CG}} \quad \cdots (6)$$

$$E_{\mathrm{BDL}} = \frac{V_{\mathrm{BDL}}}{d_{\mathrm{BDL}}} = \frac{V_{\mathrm{CG}} - V_{\mathrm{FG}}}{d_{\mathrm{BDL}}} = \frac{(1-\alpha_{\mathrm{CR}})V_{\mathrm{CG}}}{d_{\mathrm{BDL}}} = \frac{k_{\mathrm{TDL}}A_{\mathrm{TDL}}}{d_{\mathrm{TDL}}k_{\mathrm{BDL}}A_{\mathrm{BDL}} + d_{\mathrm{BDL}}k_{\mathrm{TDL}}A_{\mathrm{TDL}}}V_{\mathrm{CG}} \quad \cdots (7)$$

where k_i and d_i (i = BDL or TDL) are the dielectric constant and thickness of BDL or TDL and C_i is the capacitance of a FG/TDL/S (for i = 'TDL') or CG/BDL/FG (for i = 'BDL') determined by k_i, d_i, and the device area A_i.

Using Eqs.(5)–(7), one can easily tell how large a field will be applied across BDL and TDL, respectively, when V_{CG} is given. Through comparison between Eq. (6) and Eq.(7), one can easily recognize $E_{BDL}/E_{TDL} = k_{TDL}A_{TDL}/(k_{BDL}A_{BDL})$; hence, having BDL with a higher k-value than TDL is very important to keep E_{BDL} remaining lower than E_{TDL}. Supplementary Fig. 1c was constructed (i) by individually measuring the electrical characteristics for FG/BDL/CG (Supplementary Fig. 1a) and S/TDL/FG (Supplementary Fig. S1b) and then (ii) by mapping their applied bias to V_{CG} via Eqs.(4)-(7). With the same A_{BDL} and A_{TDL}, one can expect that higher k_{BDL} than k_{TDL} causes lower E_{BDL} than E_{TDL}, leading to lower J_{BDL} than J_{TDL} at high V_{CG} as shown in Supplementary Fig. 1c.
