# Supplementary material for: Organic flash memory on various flexible substrates for foldable and disposable electronics
Source: Nat Commun. 2017 Sep 28;8:725. doi: 10.1038/s41467-017-00805-z (PMC5620045; doi:10.1038/s41467-017-00805-z)
Supplement: Supplementary file 3 — Description of Additional Supplementary Files [file 41467_2017_805_MOESM3_ESM.pdf]

Description of Additional Supplementary Files

File Name: Supplementary Movie 1
Description: Movie shows a 6 um-thick plastic substrate with organic flash memories.

File Name: Supplementary Movie 2
Description: Movie shows an organic flash memory on a 6 um-thick plastic substrate.
